# Investigating the agronomic performance and ecological weed-suppression effects of summer leguminous green manure in dragon fruit orchards

**DOI:** 10.7717/peerj.21350

**Published:** 2026-05-25

**Authors:** Shengjian Kuang, Heng Liao, Danjun Yao, Shan Yang, Qin Zhang, Song Qin, Yanfei Liang, Aihua Zhang

**Affiliations:** 1 Soil and Fertilizer Research Institute, Guizhou Academy of Agricultural Sciences, Guiyang, China; 2 Guizhou Key Laboratory of Cultivated Land Quality, Guiyang, China; 3 Agriculture and Rural Bureau of Luodian County, Luodian, China

**Keywords:** Summer green manure, Membership function, Ecological weed control, Dragon fruit orchards

## Abstract

**Background:**

The large-scale cultivation of dragon fruit (*Selenicereus undatus*) in China faces challenges in sustainable weed management, particularly during the summer season. There is a notable lack of suitable green manure species for mountainous orchards that can effectively suppress weeds while enhancing ecological sustainability.

**Methods:**

From March to June 2019, a field trial was conducted in a mountainous dragon fruit orchard in Guizhou Province. A comprehensive evaluation of nine leguminous green manure species was performed using membership function analysis. Agronomic performance and weed-suppression effects were analyzed, with natural grass cover as a control, and correlations with green manure growth traits were examined.

**Results:**

Common bean (*Phaseolus vulgaris*), rice bean (*Vigna umbellata*), and bush bean (*Phaseolus vulgaris dwarf cv.*) achieved the highest comprehensive scores (0.71, 0.70, and 0.62, respectively). Intercropping with leguminous green manures significantly reduced weed species richness and diversity. Five species demonstrated weed control efficacy exceeding 90%: bush bean (95.98%), cowpea (*Vigna unguiculata*, 95.59%), adzuki bean (*Vigna angularis*, 92.32%), rice bean (91.16%), and common bean (90.11%). Correlation analysis indicated that rapid emergence, vigorous early growth, and high fresh biomass were significantly associated with enhanced weed suppression.

**Conclusion:**

Common bean, rice bean, and bush bean are recommended as optimal summer green manure species for weed management in mountainous dragon fruit orchards. Rapid emergence and high biomass accumulation are key traits for effective weed suppression. Their prioritized application is advised, with subsequent long-term, multilocation trials recommended to assess their comprehensive ecological and economic benefits.

## Introduction

China is the world’s largest producer of dragon fruit (*Selenicereus undatus*), with cultivation areas exceeding 66,700 hectares and continuing to expand rapidly (Chen, 2026). This large-scale production is undergoing a critical transition toward ecological sustainability, as traditional weed and soil management practices impose considerable environmental pressure (Ou et al., 2019). The challenge is particularly acute in the mountainous orchards of regions such as Guizhou and Guangxi, where prolific summer weed growth is difficult and costly to control, severely constraining industry development (Fu et al., 2016). Although green manure cultivation is recognized as an ecological practice that effectively suppresses weeds (Tursun et al., 2018; Zhang et al., 2019; Zhu et al., 2020) and improves soil quality (Cao, Zhou & Gao, 2024; Ding & Duan, 2021), existing research has focused predominantly on winter green manures (Dong et al., 2021; Liu et al., 2023; Niu et al., 2020; Zhang et al., 2021). A significant knowledge gap remains regarding the selection and evaluation of suitable summer green manure varieties, particularly those adapted to the specific conditions of mountainous orchard systems (Kuang et al., 2022).

To address this issue, this study conducted the first comprehensive field evaluation of nine leguminous green manure species in a mountainous dragon fruit orchard *via* membership function analysis. With ecological weed control as the primary objective, this research aimed to (1) identify the most promising summer green manure species adapted to local conditions with optimal weed suppression efficacy and (2) elucidate the relationships between key green manure growth characteristics and weed control effectiveness, thereby providing a scientific basis for developing low-cost, environmentally friendly weed management strategies.

## Materials & Methods

### Experimental materials

Our study examined nine leguminous green manure resources (Table 1) as suitable summer green manure options.

### Experimental design

The field experiment was conducted in 2019 in a dragon fruit orchard located in Luodian County, Guizhou Province, China. The orchard featured the ‘Zihonglong’ cultivar, which was seven years of age and in peak fruiting condition during the study. The plants were spaced 3 m between rows and 2 m within rows *via* a cement post and disc cultivation system with three plants per post. The site experiences a subtropical monsoon climate (altitude 440 m, annual precipitation 1,335 mm, mean annual temperature 19.70 °C). Soil analysis prior to sowing (0–20 cm depth, red soil) revealed the following properties: pH, 6.30; organic matter 22.09 g kg^−^^1^, total N 1.40 g kg^−^^1^, available N 113 mg kg^−^^1^, total P 0.81 g kg^−^^1^, available P 93.2 mg kg^−^^1^, total K 2.27 g kg^−^^1^, available K 98 mg kg^−^^1^.

A randomized complete block design with three replications was employed. Each block contained plots for all nine green manure treatments and one natural grass cover control, which were randomly arranged. The individual plot dimensions were 12 m × 3 m, resulting in a total of 30 plots. Green manures were sown on March 11, 2019. The field underwent complete manual weeding five days prior to sowing to remove all visible weed plants and residues, followed by shallow tillage and levelling. Sowing was conducted in rows with 50 cm spacing. The seeding rate was 22.5 kg ha^−1^ for alfalfa and 30 kg ha^−1^ for all other species. No basal or top-dressing fertilizers were applied to the green manures.

The irrigation and fertilization management of the dragon fruit trees followed local conventional practices. Irrigation relies primarily on natural rainfall. Fertilization consisted of a winter basal application and four seasonal top-dressings. The basal fertilizer, comprising 10.0 kg of well-decomposed sheep manure per post, was applied using a ring-trench method during dormancy. Top dressing with a balanced NPK compound fertilizer (N-P_2_O_5_-K_2_O = 15-15-15) at a rate of 0.6 kg per post was carried out in mid-April, late May, early July, and early September.

**Table 1:** Legume green manure accessions evaluated in the study.

Accession No.	Resource Name	Genus	Species	Origin
X1	Soybean	Glycine	*Glycine max*	Locally collected
X2	Mung bean	Vigna	*Vigna radiata*	Locally collected
X3	Peanut	Arachis	*Arachis hypogaea*	Market-purchased
X4	Common bean	Phaseolus	*Phaseolus vulgaris*	Locally collected
X5	Cowpea	Vigna	*Vigna unguiculata*	Market-purchased
X6	Alfalfa	Medicago	*Medicago sativa*	Market-purchased
X7	Rice bean	Vigna	*Vigna umbellata*	Locally collected
X8	Bush bean	Phaseolus	*Phaseolus vulgaris dwarf cv.*	Locally collected
X9	Adzuki bean	Vigna	*Vigna angularis*	Locally collected

### Measurements and Methods

#### Green manure growth traits and nutrient contents

Phenological stages (emergence, budding, initial flowering, and full bloom) were recorded for each species. At the full bloom stage, a 1 m^2^ quadrat was randomly selected per plot to harvest aboveground biomass to determine the fresh yield and measure the plant height. The samples were oven-dried for further analysis. As different species reached full bloom at different times, harvests occurred on two dates: soybean, mung bean, common bean, cowpea, bush bean, and adzuki bean were harvested on May 13, 2019; peanut, alfalfa, and rice bean were harvested on June 13, 2019. The total nitrogen content of the plants was determined using the Kjeldahl method, the total phosphorus content was determined *via* molybdenum blue colorimetry, the total potassium content was determined *via* flame photometry, and the total carbon content was determined *via* the potassium dichromate method.

#### Evaluation of green manure agronomic performance

To enable a multidimensional comparison, a membership function analysis was applied (Yao et al., 2009). For traits positively correlated with comprehensive performance, the following formula was used:

*Z*_*ij*_ =(*X*_*ij*_ − *X*_*imin*_)/(*X*_*imax*_ − *X*_*imin*_).

For traits negatively correlated with comprehensive performance (emergence days and dry-to-fresh ratio), the formula was:

*Z*_*ij*_ =1−[(*X*_*ij*_ − *X*_*imin*_)/(*X*_*imax*_ − *X*_*imin*_)].

Here, *Z*_*ij*_ is the membership function value of trait *j*-th for variety *i*-th; *X*_*max*_ and *X*_*min*_ are the maximum and minimum values of trait *j*-th among all varieties, respectively. The average membership function value across all traits for each variety was calculated as its final comprehensive score. To avoid bias from any single trait in this preliminary screening study, equal weights were assigned to all evaluated traits, a method commonly used in similar germplasm resource evaluations (Li et al., 2026).

Twelve evaluation traits were selected based on the technical requirements for understory weed control and soil improvement in orchards, combined with green manure growth characteristics (Zhang et al., 2022) (Table 2). All traits were quantitative except for the “non-climbing rate,” which was a qualitative assessment. Climbing behavior was observed and rated on a 0–100% scale: varieties with completely upright, non-climbing stems were assigned 100%; varieties with strong twining or tendril-climbing ability were assigned 0%; varieties with intermediate behavior received scores between 0% and 100%. In this study, bush bean was an upright type; cowpea and common bean were strong climbing types.

**Table 2:** Establishment of evaluation criteria.

Code	Evaluation indicator	Attribute	Rationale for requirements
K1	Emergence time (d)	Negative	Short emergence time with rapid ground coverage
K2	Plant height in first month (cm)	Positive	Fast seedling growth enabling quick ground coverage
K3	Maximum plant height (cm)	Positive	Lush plant growth
K4	Ground coverage duration (d)	Positive	Prolonged coverage duration from emergence to full bloom for effective weed suppression
K5	Flowering spike duration (d)	Positive	Prolonged coverage duration from bud formation to full bloom for effective weed suppression
K6	Fresh biomass yield (g m^−^^2^)	Positive	High biomass production with elevated nutrient return potential
K7	Dry-to-fresh ratio	Negative	Tender plant tissue facilitating rapid decomposition
K8	C/N ratio	Positive	High decomposability when incorporated
K9	Non-climbing rate	Positive	Minimal interference with orchard tree growth
K10	Returnable nitrogen content (g m^−^^2^)	Positive	High nitrogen recycling capacity
K11	Returnable phosphorus content (g m^−^^2^)	Positive	Elevated phosphorus return potential
K12	Returnable potassium content (g m^−^^2^)	Positive	Substantial potassium restitution capacity

**Notes.**

When the C/N ratio reaches 25, it optimally facilitates microbial breakdown of plant residues in soil and improves nutrient release efficiency. Given that the examined green manure resources had C/N ratios ranging from 13 to 22, this statistic was selected as a positive evaluation indicator.

#### Weed survey in the dragon fruit orchard

Weed surveys were conducted synchronously when each green manure species reached its full bloom stage. At this point, green manure biomass and competitive effects are maximal, and summer annual weeds are also at a vigorous growth or early flowering stage, facilitating species identification and providing an accurate reflection of suppression effects. One 1 m^2^ quadrat was randomly placed in each plot to inventory weed species and count individuals.

#### Weed diversity and control efficacy calculations

Species richness (S) was defined as the total number of weed species in the community. Abundance (*Pi*) was calculated using the Berger–Parker formula:

*P*_*i*_ = *N*_*i*_/ *N*

where: *N*_*i*_ number of individuals of the *i*-th weed species

N = total number of weed individuals in the quadrat (*N* =∑*N*_*i*_)

Species diversity (*H*) was assessed using the Shannon-Wiener diversity index, which was then transformed into its exponential form exp(*H′*) to represent effective species number for more intuitive interpretation of diversity (Jost, 2006):

*H′* = −∑*P*_*i*_× ln(*P*_*i*_)

exp(*H′*) = *e* ^*H*′^

Evenness (*J*) was calculated using Heip’s evenness index based on exp(*H′*):

*E*_*Heip*_ =(*e* ^*H*^′ − 1)/(*S*−1)

Dominance Concentration (*C*) was determined using Simpson’s formula:

*C* = ∑*P*_*i*_^2^

Weed Control Efficacy (%) (Luan, 2015) was calculated as:

Control Efficacy (%) = [(Weed density in control – Weed density in treatment)/Weed density in control] ×100.

#### Observational assessment of intercropping effects on dragon fruit growth

The primary objective of this trial was the preliminary screening of green manures for weed suppression. Therefore, quantitative measurements of dragon fruit yield and quality were not included. However, systematic field observations throughout key growth stages (spring shoot, flowering, and fruit expansion) were conducted to note any visually discernible adverse effects on the main crop compared to the natural grass control.

### Statistical analysis

The membership function values, weed community indices, and control efficacy values were calculated using Microsoft Excel 2016. Differences in weed community indices (richness, diversity, evenness, dominance concentration) among the treatments were analyzed using GraphPad Prism 10.6. As the data for weed richness and diversity indices violated assumptions of normality (Shapiro–Wilk test) and homogeneity of variance (Levene’s test for richness and evenness), non-parametric Kruskal–Wallis H tests were used to assess overall differences. Where significant, Dunn’s tests with Bonferroni correction were applied for *post hoc* pairwise comparisons. Spearman’s rank correlation analysis was performed using SPSS 20.0 to examine the relationships between the green manure growth traits and weed parameters. Graphs were created using OriginPro 2021.

## Results

### Agronomic trait performance and comprehensive evaluation of leguminous green manures

As shown in Table 3, the emergence time of the green manures ranged from 7 to 18 days, with bush bean being the fastest and peanut the slowest. The common bean exhibited the most vigorous early growth, reaching a height of 98.3 cm in the first month and the maximum height at full bloom (113.7 cm). Rice bean provided the longest duration of ground cover (84 days), whereas mung bean had the shortest duration (50 days). Bush bean produced the highest fresh biomass yield (1,943.33 g m^−2^), whereas alfalfa yielded the lowest (725.00 g m^−2^).

**Table 3:** Performance characteristics of nine leguminous green manure resources.

Accession No.	K1	K2	K3	K4	K5	K6	K7	K8	K9	K10	K11	K12
X1	12	20.0	65.5	51	30	1,641.67	25.12	13.14	100	12.83	1.43	7.02
X2	13	18.6	66.0	50	25	826.67	21.38	16.72	100	4.20	0.75	4.06
X3	18	8.7	48.4	76	35	1,208.33	22.94	21.08	100	5.33	0.89	6.12
X4	8	98.3	113.7	55	30	1,850.00	18.71	21.76	20	7.02	1.42	8.01
X5	12	25.0	99.5	51	25	1,693.33	19.33	15.81	20	8.56	1.13	7.61
X6	15	15.9	54.5	79	55	725.00	19.35	16.69	100	3.57	0.72	4.08
X7	10	18.2	87.0	84	50	1,788.33	24.29	20.31	95	8.97	1.46	6.35
X8	7	37.2	59.3	56	20	1,943.33	19.61	18.55	100	8.49	1.47	6.04
X9	13	17.2	35.6	50	25	880.00	22.89	14.86	100	5.55	0.71	2.73

The comprehensive evaluation scores based on membership function analysis are presented in Table 4. Common bean, rice bean, and bush bean ranked highest, with scores of 0.71, 0.70, and 0.62, respectively.

**Table 4:** Membership function values and ranking of various traits of leguminous green manure resources.

Accession No.	K1	K2	K3	K4	K5	K6	K7	K8	K9	K10	K11	K12	Mean	Ranking
X1	0.55	0.13	0.38	0.03	0.29	0.75	0.00	0.00	1.00	1.00	0.96	0.81	0.49	4
X2	0.45	0.11	0.39	0.00	0.14	0.08	0.58	0.42	1.00	0.07	0.06	0.25	0.30	8
X3	0.00	0.00	0.16	0.76	0.43	0.40	0.34	0.92	1.00	0.19	0.23	0.64	0.42	6
X4	0.91	1.00	1.00	0.15	0.29	0.92	1.00	1.00	0.00	0.37	0.94	1.00	0.71	1
X5	0.55	0.18	0.82	0.03	0.14	0.79	0.90	0.31	0.00	0.54	0.56	0.92	0.48	5
X6	0.27	0.08	0.24	0.85	1.00	0.00	0.90	0.41	1.00	0.00	0.02	0.25	0.42	7
X7	0.73	0.11	0.66	1.00	0.86	0.87	0.13	0.83	0.94	0.58	0.99	0.69	0.70	2
X8	1.00	0.32	0.30	0.18	0.00	1.00	0.86	0.63	1.00	0.53	1.00	0.63	0.62	3
X9	0.45	0.10	0.00	0.00	0.14	0.13	0.35	0.20	1.00	0.21	0.00	0.00	0.22	9

### Weed community composition in orchards

The survey identified 14 annual weed species belonging to 14 genera and 11 families (Table 5). *Asteraceae* (three species) and *Poaceae* (two species) were the most represented families. *Eleusine indica* was the most dominant species, with the highest density (62.37 plants m^−2^) and a frequency of occurrence of 1.00 (present in all the sampled quadrats). *Commelina communis* was the second most dominant species (12.73 plants m^−2^) and was the most common species (0.87).

**Table 5:** Weed community composition in dragon fruit orchards.

Family	Genus	Species	Density (plants/m^2^)	Frequency
*Asteraceae*	*Galinsoga*	*Galinsoga parviflora*	7.43	0.63
	*Bidens*	*Bidens pilosa*	0.70	0.10
	*Crassocephalum*	*Crassocephalum crepidioides*	0.07	0.07
*Poaceae*	*Eleusine*	*Eleusine indica*	62.37	1.00
	*Digitaria*	*Digitaria sanguinalis*	3.53	0.27
*Commelinaceae*	*Commelina*	*Commelina communis*	12.73	0.87
*Brassicaceae*	*Brassica*	*Brassica juncea var.*	7.30	0.33
*Oxalidaceae*	*Oxalis*	*Oxalis corniculata*	0.97	0.30
*Amaranthaceae*	*Amaranthus*	*Amaranthus retroflexus*	1.13	0.27
*Euphorbiaceae*	*Acalypha*	*Acalypha australis*	0.57	0.13
*Cyperaceae*	*Cyperus*	*Cyperus rotundus*	0.33	0.10
*Caryophyllaceae*	*Myosoton*	*Myosoton aquaticum*	0.17	0.07
*Scrophulariaceae*	*Veronica*	*Veronica polita*	0.57	0.07
*Convolvulaceae*	*Ipomoea*	*Ipomoea purpurea*	0.10	0.07

#### Impact of leguminous green manure intercropping on weed community characteristics

The results of the Kruskal–Wallis tests revealed a significant overall effect of treatment on weed species richness (*H* = 19.43, *P* = 0.022) and diversity (*H* = 17.77, *P* = 0.038) but not on evenness (*P* = 0.455) or dominance concentration (*P* = 0.120) (Table 6). *Post hoc* Dunn’s tests revealed no statistically significant pairwise differences between treatments for any index after Bonferroni correction.

**Table 6:** Results of significance analysis on differences in weed community characteristics among different green manure treatments.

Treatment	Richness	Diversity	Evenness	Species dominance
K-W test H value	19.43	17.77	8.81	14.05
K-W test *P* value	0.022	0.038	0.455	0.120
Dunn’s *post hoc* pairwise comparison results	*P* > 0.05	*P* > 0.05	*P* > 0.05	*P* > 0.05

**Notes.**

Dunn’s *post hoc* pairwise comparison results presented in the table are the *p*-values adjusted by the Bonferroni correction.

**Figure 1:**
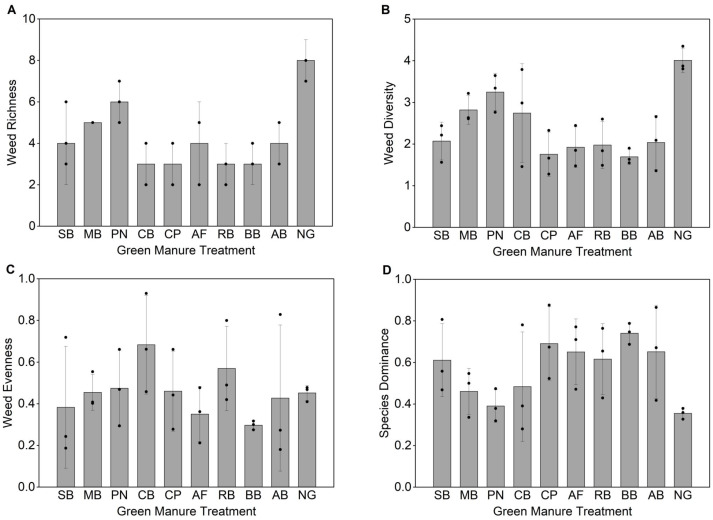
Weed species richness under different green manure treatments. Bars represent the mean ± standard deviation; individual data points (*n* = 3) are overlaid. Treatment abbreviations: SB, Soybean; MB, Mung bean; PN, Peanut; CB, Common bean; CP, Cowpea; AF, Alfalfa; RB, Rice bean; BB, Bush bean; AB, Adzuki bean; NG, Natural Grass Cover.

Visual inspection of the data (Fig. 1) revealed that, compared with the natural grass cover, all the green manure treatments reduced weed richness. Treatments involving common bean, cowpea, rice bean, and bush bean were most effective, lowering species richness to 3.0, a 62.5% reduction from the control (8.0). Weed diversity was also lower under all the green manure treatments. Notably, the bush bean and cowpea treatments resulted in the lowest diversity indices (1.69 and 1.76, decreases of 57.9% and 56.1%, respectively), but the highest dominance concentration values (0.74 and 0.69, increases of 105.6% and 91.7%, respectively) significantly exceeded those of the control (0.36). This is a key finding, indicating that although these two types of green manures strongly suppressed overall weed richness and diversity, they may have selectively driven the weed community toward concentration in a few dominant species, thereby altering the competitive structure of the community.

#### Weed control efficacy of green manure cover

The weed control efficacy varied among the green manure species (Table 7). The efficacy exceeded 90% for bush bean (95.98%), cowpea (95.59%), adzuki bean (92.32%), rice bean (91.16%), and common bean (90.11%). Peanut exhibited the lowest control efficacy (54.12%).

**Table 7:** Weed control efficiency of leguminous green manures in dragon fruit orchards (%).

Weed species	Soybean	Mung bean	Peanut	Common bean	Cowpea	Alfalfa	Rice bean	Bush bean	Adzuki bean
*Myosoton aquaticum*	33.33	100.00	100.00	100.00	100.00	100.00	100.00	100.00	100.00
*Amaranthus retroflexus*	100.00	−36.36	45.45	100.00	100.00	81.82	100.00	100.00	100.00
*Bidens pilosa*	90.00	100.00	100.00	0.00	100.00	100.00	100.00	100.00	100.00
*Brassica juncea var.*	86.79	15.09	98.11	100.00	100.00	93.40	100.00	100.00	100.00
*Digitaria sanguinalis*	100.00	93.41	100.00	100.00	97.80	100.00	100.00	94.51	97.80
*Eleusine indica*	48.66	52.32	62.35	94.62	81.91	−1.22	54.52	77.02	72.37
*Galinsoga parviflora*	100.00	66.67	−175.76	90.91	96.97	−100.00	90.91	90.91	63.64
*Veronica polita*	100.00	100.00	100.00	93.75	100.00	100.00	100.00	100.00	100.00
*Acalypha australis*	100.00	100.00	−42.86	100.00	100.00	100.00	100.00	100.00	100.00
*Cyperus rotundus*	100.00	20.00	80.00	100.00	100.00	100.00	100.00	100.00	100.00
*Commelina communis*	−40.38	−40.38	−9.62	98.08	61.54	9.62	30.77	86.54	69.23
*Crassocephalum crepidioides*	100.00	100.00	100.00	100.00	100.00	0.00	100.00	100.00	100.00
*Ipomoea purpurea*	100.00	50.00	100.00	100.00	100.00	100.00	100.00	100.00	100.00
*Oxalis corniculata*	84.21	94.74	100.00	84.21	100.00	100.00	100.00	94.74	89.47
Mean	78.76	58.25	54.12	90.11	95.59	63.11	91.16	95.98	92.32

#### Correlation between green manure growth traits and weed parameters

Spearman correlation analysis (Table 8) revealed significant relationships. The green manure emergence time was strongly positively correlated with weed richness and significantly negatively correlated with control efficacy. Conversely, first-month plant height exhibited a strong negative correlation with weed richness. The full-bloom plant height and fresh biomass yield were also significantly negatively correlated with weed richness.

**Table 8:** Correlations between leguminous green manure growth traits, weed community characteristics, and control efficiency.

Growth traits	Richness	Diversity	Evenness	Species dominance	Weed control efficacy
Emergence time	0.863^**^	0.462	−0.067	−0.418	−0.714^*^
Plant height in first month	−0.612^**^	−0.321	0.202	0.292	0.567
Maximum plant height	−0.427^*^	−0.165	0.297	0.083	0.200
Fresh biomass yield at full bloom	−0.718^*^	−0.333	0.133	0.318	0.633

**Notes.**

* *P* < 0.05.

** *P* < 0.01.

#### Observational assessment of intercropping effects on dragon fruit growth

Importantly, the primary objective of this trial was the preliminary screening of green manures for weed suppression. Therefore, quantitative measurements of dragon fruit yield and quality were not included. However, systematic field observations throughout key growth stages (spring shoot, flowering, and fruit expansion) revealed no visually discernible symptoms of growth inhibition or nutrient deficiency in dragon fruit plants under any of the green manure intercropping treatments compared with the natural grass control. This provides preliminary visual evidence for the field compatibility of this intercropping practice.

## Discussion

### Agronomic performance and selection of summer green manures

This study successfully identified common bean, rice bean, and bush bean as superior summer green manure candidates for mountainous dragon fruit orchards, which aligns with the potential of utilizing specific legume traits for green manure applications (Tani et al., 2017). The selection criteria focused on rapid growth, high biomass, effective ground cover, and suitability for orchard management (Gong et al., 2021; Kuang et al., 2022; Zhang et al., 2022), providing a practical framework for local adoption. Future breeding efforts targeting these traits in leguminous crops are warranted.

### Modifications of weed community structure

Consistent with other orchard studies (Chen et al., 2024; Wang et al., 2022; Zhang et al., 2018), Poaceae and Asteraceae species, particularly *Eleusine indica* and *Commelina communis*, were the most prevalent weeds. A reduction in overall weed richness and diversity under green manure cover is a common finding, similar to results reported in coffee plantations (Fu et al., 2021). In contrast, some studies have reached opposite conclusions. For example, intercropping white clover in pomelo orchards was reported to effectively control weed growth, stabilize the weed community in the orchard without a prominent dominant species and reduce the likelihood of severe infestation (Zhang et al., 2018; Zhang et al., 2019). However, the concurrent increase in weed dominance concentration, especially under bush bean and cowpea, is a critical observation. This suggests that while these highly competitive green manures exert broad suppression, they may selectively favor the persistence or relative increase of a few resilient species, potentially simplifying the community structure.

This study demonstrates that the broad-spectrum suppression from green manure acts as a selective pressure, enriching competitive weeds with specific adaptations. For instance, deep-rooted *Eleusine indica* and shade-tolerant *Commelina communis* were able to escape inhibition, resulting in increased dominance. This highlights that green manure intercropping is not a standalone solution but should be integrated into a multitiered weed management strategy that may include targeted physical or biological control for persistent weeds. The goal of ecological management is to regulate, not eradicate, weed communities while preserving biodiversity (MacLaren et al., 2020).

### Efficacy and limitations in weed control

The results of this study indicate that five leguminous green manure species achieved an average weed control efficacy exceeding 90%, indicating highly significant weed suppression. This finding aligns with previous research, where the use of green manure in orchards typically results in weed control rates of 60–80% or higher (Liang, Zhou & Jiang, 2021; Luan, 2015; Wang, Li & Zhang, 2017), with some studies reporting reductions in weed biomass of as much as 99% (Menalled et al., 2023). Furthermore, research has shown that mixed sowing of cover crops provides better weed suppression than single-species planting (McKenzie-Gopsill et al., 2022), offering valuable insights for future in-depth investigations.

This study also revealed that green manures exhibited limited control efficacy against certain pernicious weeds, which has dual ecological implications. The advantage is that future weed management efforts can focus more on a narrower set of targets, whereas the risk is that simplified weed community structures may result in reduced ecological resilience, making control more challenging if dominant weeds experience outbreaks. Therefore, in practice, attention must be given to the potential for green manures to inadvertently select for and reinforce specific hard-to-control weeds. Additionally, peanuts exhibit relatively poor weed suppression, primarily due to their prostrate growth habit, which leads to slow early establishment and failure to form effective ground cover rapidly, thereby creating a “time window” for weed emergence.

### Key traits underlying weed suppression

This study confirmed that key growth traits of leguminous green manures, such as emergence time, plant height, and biomass at full bloom, are significantly correlated with weed richness and control efficacy. Varieties that emerge faster, exhibit more vigorous early growth, and produce greater fresh biomass demonstrated a greater ability to reduce weed species diversity and enhance control effectiveness. This finding is consistent with previous research: the competitive ability of cover crops is closely linked to their biomass and canopy development speed, with rapid early canopy formation being crucial for weed suppression (Brennan & Smith, 2005; Gaudet & Keddy, 1988). Early sowing often enhances suppressive effects, such as shading, by increasing plant biomass (Akbari et al., 2019; Haring & Hanson, 2022; Teasdale & Mohler, 1993). The underlying mechanisms primarily involve resource competition (Chen et al., 2024; Fu et al., 2021; Mirsky et al., 2013), physical obstruction (Fernando & Shrestha, 2023; Teasdale & Mohler, 2000), and potential allelopathic effects (Dhima et al., 2009; Gavazzi et al., 2010; Khamare, Chen & Marble, 2022). Together, these mechanisms not only suppress the growth of existing weeds but also influence the soil seed bank, reducing weed seed germination (Liu et al., 2010; Melander, Rasmussen & Olesen, 2020; Sun, Lu & Xin, 2011).

From a practical perspective, this study provides clear varietal selection criteria for ecological weed control: priority should be given to green manure varieties that exhibit rapid emergence, fast first-month height growth, and high biomass at full bloom, such as bush bean and cowpea, as demonstrated in this study. Future research could explore optimized approaches, such as the mixed sowing of green manures, to develop more stable and sustainable ecological management systems for orchards.

### Limitations and future research directions

As a preliminary screening study was conducted at a single location over one growing season, the broader applicability of these findings requires validation through multilocation, multiyear trials. However, it provides directly testable candidate varieties and evaluation methods for similar ecological regions. This study focused primarily on the growth and weed suppression traits of the green manures themselves. A critical next step is to initiate long-term, system-level experiments to comprehensively evaluate the impact of these intercropping systems on dragon fruit yield, fruit quality, soil health indicators, and overall economic profitability, thereby completing the transition from “variety screening” to “system benefit assessment.” While green manure cover can significantly suppress overall weeds, it may also increase the dominance of a few resilient species. Therefore, in practice, it is essential to combine “broad-spectrum green manure cover” with “targeted control of persistent weeds” to develop an integrated management strategy. Although measurements taken at the full-bloom stage of each variety align more closely with agricultural practices, they may introduce temporal confounding. Future studies could optimize this by standardizing monitoring time points.

## Conclusion

This study identified common bean, rice bean, and bush bean as highly effective summer green manure species for ecological weed management in mountainous dragon fruit orchards, with weed control efficacy exceeding 90%. Rapid emergence and the accumulation of high biomass were identified as key phenotypic traits predictive of strong weed suppression capacity. For practical application, a seeding rate of about 30 kg ha^−1^ is suggested for species such as bush bean and common bean. Future research should prioritize long-term, multilocation trials to verify the stability and adaptability of these varieties, explore the potential benefits of mixtures of green manure species, and systematically evaluate the long-term ecological and agronomic impacts of this intercropping system on orchard productivity and sustainability.

##  Supplemental Information

10.7717/peerj.21350/supp-1 Supplemental Information 1 Raw Data

## Additional Information and Declarations

### Data Availability

The following information was supplied regarding data availability: The raw data is available in the Supplemental File.
